# Diagnostics and the neglected tropical diseases roadmap: setting the agenda for 2030

**DOI:** 10.1093/trstmh/traa118

**Published:** 2020-11-09

**Authors:** Ashley A Souza, Camilla Ducker, Daniel Argaw, Jonathan D King, Anthony W Solomon, Marco A Biamonte, Rhea N Coler, Israel Cruz, Veerle Lejon, Bruno Levecke, Fabricio K Marchini, Michael Marks, Pascal Millet, Sammy M Njenga, Rahmah Noordin, René Paulussen, Esvawaran Sreekumar, Patrick J Lammie

**Affiliations:** Neglected Tropical Diseases Support Center, Task Force for Global Health, Atlanta, GA 30030, USA; Consultant, World Health Organization, Geneva, Switzerland; Department of Control of Neglected Tropical Diseases, World Health Organization, Geneva, Switzerland; Department of Control of Neglected Tropical Diseases, World Health Organization, Geneva, Switzerland; Department of Control of Neglected Tropical Diseases, World Health Organization, Geneva, Switzerland; Drugs and Diagnostics for Tropical Diseases, San Diego, CA 92111, USA; Center for Global Infectious Disease Research, Seattle Children’s Research Institute, Seattle, WA, USA; National School of Public Health, Instituto de Salud Carlos III, Madrid, Spain; Institut de Recherche pour le Développement, University of Montpellier, Montpellier, France; Department of Virology, Parasitology and Immunology, Ghent University, Faculty of Veterinary Medicine, Salisburylaan 133, B-9820 Merelbeke, Belgium; Carlos Chagas Institute, Fiocruz Parana, Curitiba, Parana, Brazil; Clinical Research Department, Faculty of Infectious and Tropical Diseases, London School of Hygiene & Tropical Medicine, London, WC1E 7HT, UK; Laboratoire de Parasitologie, Université de Bordeaux, Bordeaux, France; Kenya Medical Research Institute, Nairobi, Kenya; Institute for Research in Molecular Medicine, Universiti Sains Malaysia, Pengang, Malaysia; Mondial Diagnostics, Amsterdam, the Netherlands; Molecular Virology Laboratory, Rajiv Gandhi Centre for Biotechnology, Thiruvananthapuram, Kerala, India; Neglected Tropical Diseases Support Center, Task Force for Global Health, Atlanta, GA 30030, USA

**Keywords:** diagnostics, neglected tropical diseases, target product profiles, WHO

## Abstract

Accurate and reliable diagnostic tools are an essential requirement for neglected tropical diseases (NTDs) programmes. However, the NTD community has historically underinvested in the development and improvement of diagnostic tools, potentially undermining the successes achieved over the last 2 decades. Recognizing this, the WHO, in its newly released draft roadmap for NTD 2021–2030, has identified diagnostics as one of four priority areas requiring concerted action to reach the 2030 targets. As a result, WHO established a Diagnostics Technical Advisory Group (DTAG) to serve as the collaborative mechanism to drive progress in this area. Here, the purpose and role of the DTAG are described in the context of the challenges facing NTD programmes.

## Introduction

The 2012 WHO neglected tropical diseases (NTDs) roadmap and subsequent London declaration on NTDs galvanised international support for the control and elimination of NTDs by 2020.^1^ Renewed and expanded drug donations and financial commitments created a favourable environment for the scaling up of control and elimination programmes across all NTD-endemic geographies. Today, the scope of global programmes targeting NTDs is unprecedented, reaching more than one billion people annually and an increasing number of countries are achieving elimination goals.^2^ Despite enormous successes, NTD programmes have not yet met their original and ambitious 2020 targets, necessitating the development of a new NTD roadmap for 2021–2030.

The 2021–2030 roadmap is meant to serve as a guiding document to facilitate alignment across the global community of NTD partners by defining global targets to guide the delivery of programmes across the 20 WHO-targeted NTDs.^3^ It is also intended to serve as a policy and advocacy document, drawing attention to the key challenges facing NTD programmes and encouraging continued commitment from the global community of partners. Among the priority areas identified in the roadmap, the following four are considered to have the most critical gaps impeding progress towards the 2030 targets across multiple NTDs:

- Diagnostics.
- Monitoring and evaluation (M&E).
- Access and logistics.
- Advocacy and funding.

Accurate, reliable and affordable diagnostic tools are an essential requirement for NTD programmes. They support individual-level treatment choices, inform population-level decisions on changing treatment frequency or stopping mass treatment, enable disease surveillance and allow for confidence in validating or verifying elimination or certifying eradication. Simply put, diagnostic tools are the drivers of M&E and are critical to documenting impact. Although classical clinical and microscopic techniques are often adequate for mapping disease distribution and for monitoring the progress of most NTD interventions, the need for improved diagnostics comes into much sharper focus as infection prevalence declines and elimination or eradication becomes a possibility.^4,5^ The NTD community has traditionally underinvested in the development and improvement of diagnostic tools, potentially undermining the success that has been achieved.

As an indication of the magnitude of the need for new diagnostic tools, in the WHO 2030 Roadmap, 13 disease communities listed diagnostics among their top three critical actions to reach the 2030 targets and 18 diseases indicated that diagnostics require critical action to reach the 2030 targets.^3^ Where existing diagnostics are in theory sufficient for programmatic needs, the availability of tests at the correct levels of the health system may be a challenge. Both new tests and test formats are now needed to achieve the 2021–2030 roadmap targets.

Identifying diagnostics as a priority area provides formal acknowledgment of the critical role these tools play in monitoring and evaluating NTD programmes and reinforces the necessity for the NTD community to allocate resources for diagnostic tools to achieve the 2030 targets.

## Development of the Diagnostic Technical Advisory Group

In 2009, the WHO Department of Control of Neglected Tropical Diseases established an M&E working group to engage researchers and programme implementers in the development of standardised tools to strengthen M&E frameworks across all NTDs. Reports from the field indicated that multiple NTD programmes were facing challenges with diagnostic tools that were lacking in sensitivity and specificity, and which were unreliable or inaccessible. As a result, the M&E working group recommended that a dedicated diagnostics group be developed, a recommendation that was endorsed by the Strategic Technical Advisory Group.^6^ The Diagnostics Technical Advisory Group (DTAG) was then established in anticipation of the 2030 roadmap and to serve as the WHO's mechanism for collaborative development of new diagnostic tools.^7^

The DTAG was charged with responsibility to:

- Review and prioritise diagnostics needs for NTD programmes.
- Define the use cases (i.e. the programmatic context in which the test is used) and target product profiles (TPPs) for the necessary diagnostic tools.
- Link with key partners to support test development and validation.
- Provide the WHO with guidance on the utility of new tools to support the control and elimination of NTDs.

DTAG members were selected to represent a broad range of expertise in test development, M&E and programme implementation, as well as disease-specific expertise. Recognising that such a small membership would struggle to provide the in-depth expertise required to address all 20 NTDs, development of time-limited subgroups was recommended to provide more specific expertise.

The development of the DTAG provides formal WHO recognition of the extent of the diagnostics challenge in achieving the 2030 targets and represents the first large-scale, coordinated strategy to address it. It will provide an essential connection between the WHO, the donor community and product developers to ensure that the needs of the programmes will be met.

## Defining the agenda

At the inaugural meeting of the DTAG in October 2019, it was noted that the vast majority of NTD programmes rely upon diagnostic tools to facilitate interventions at the individual level or public health actions at the population level. Consultations across the NTD community provided a landscape analysis that guided discussions at the meeting. Limited resources require prioritisation of the most urgent needs, bearing in mind that all diagnostic needs across all 20 NTDs will have to be addressed over time.

Prioritisation of needs is not a straightforward task. The exercise required a defined set of criteria upon which to rank needs and their urgency. To provide a transparent basis for establishing initial priorities, DTAG members employed an algorithm that considered the current state of the programme targeting a specific NTD, and whether a new diagnostic tool is needed to inform decision-making for existing programmes or to achieve 2030 targets. If a new diagnostic tool is necessary, either to provide essential support for an ongoing programme or to meet 2030 targets, then the priority is considered to be high. NTDs that lack a broadly accepted public health strategy and which are not addressed currently by a global programme were acknowledged to have important needs for investment in research, but were not considered to represent critical diagnostic priorities at this stage. Prioritisation exercises will be revisited on an annual basis to keep designated priorities aligned with evolving programme strategies and needs.

For NTDs for which preventive chemotherapy forms an important part of the public health response, the need for better surveillance tools is cross-cutting. For lymphatic filariasis, the introduction of a triple-drug therapy comprising ivermectin, diethylcarbamazine and albendazole may reduce the number of rounds of mass drug administration required, thus reducing the utility of current antigen tests for stopping decisions based on transmission assessment surveys. For onchocerciasis, the performance of existing tests is inadequate in low prevalence settings, and for soil-transmitted helminths and schistosomiasis, the dependence on the collection of stool and urine samples presents both logistical and laboratory challenges. For diseases where case management is critical, improved point-of-care rapid diagnostic tests and high quality molecular tests are urgently needed. The new focus on integrated management of skin diseases, as outlined in the 2030 roadmap, would be strengthened by the deployment of multiplex tests that support differential diagnosis; these tests will need to address the additional challenge of common and standardised sample processing and target concentration/enrichment process. Specific needs for high priority use cases (defined using the approach described above) are summarised in Table 1.

**Table 1.** DTAG prioritisation of diagnostic needs and 2030 roadmap targets

Preventive chemotherapy diseases	2030 roadmap target	Is absence of a test hampering programmatic decision-making?	Is absence of test threatening 2030 targets?	Additional comments
Onchocerciasis	Elimination of transmission	Yes	Yes	Entomological and serological tools needed to demonstrate interruption of transmission
		Starting MDA in low-endemic areas and stopping MDA	Tools needed for postverification surveillance	
Lymphatic filariasis	Elimination as a public health problem	Yes	Yes	A test needed for viable adult worms would strengthen MDA stopping decisions, particularly in the context of IDA
			Tools needed for postvalidation surveillance	The cross-reactivity of FTS with *Loa loa* creates challenges in coendemic areas. Accelerating MDA with IDA requires a different impact assessment approach to TAS
Trachoma	Elimination as a public health problem	No	No	Postvalidation surveillance would be enhanced by a diagnostic tool that does not rely on clinical signs
Soil-transmitted helminthiases	Elimination as a public health problem	Yes	Yes	Current tools do not reliably detect *Strongyloides*
				Faecal samples are suboptimal for programme M&E, especially in low prevalence settings
Schistosomiasis	Elimination as a public health problem	Yes	Yes	Surveillance tools will help support the drive toward elimination
		Better tools needed to measure progress toward morbidity targets	Better tools for low prevalence settings are needed	
Yaws	Eradication	No	No	Tests needed for rapid detection of resistance. Automated high-throughput tests for serosurveillance and certification. Ideally, serological tests that can differentiate between yaws and syphilis for diagnosis in adults
Case management diseases	2030 roadmap target	Is there an established intervention strategy?	Is absence of test threatening 2030 targets?	Additional comments
Human African trypanosomiasis *Trypanosoma brucei rhodesiense* (*rHAT*), *T. brucei gambiense* (*gHAT*)	gHAT: elimination of transmission	Yes	Yes (rHAT)	High throughput testing would enhance postelimination surveillance
	rHAT: elimination as a public health problem	Tests needed to guide treatment decisions for gHAT		
Cutaneous leishmaniasis	Control	Yes	Yes	A POC test is needed for cutaneous leishmaniasis
Dengue/chikungunya/Zika virus disease	Control	Yes	Yes	Development of a combination RDT test is needed to support differential diagnosis and clinical management
Mycetoma, chromoblastomycosis and other deep mycoses	Control	Yes	Yes	RDT needed for early case detection
Buruli ulcer	Control	Yes	Yes	RDT needed to confirm diagnosis
				Digital microscopy/cell phone imaging could be a valuable technology to improve integrated management of skin disease
Leprosy	Elimination of transmission	Yes	Yes	Improved POC tests needed for implementation of postexposure prophylaxis and surveillance
Chagas disease	Elimination as a public health problem	Yes	Yes	Digital microscopy/cell phone imaging could be a valuable (cross-cutting) technology
		RDT needed to detect infection and for treatment response	RDT needed for congenital Chagas and discrete typing units	Automatic tools needed for screening (e.g. blood banks)
Visceral leishmaniasis	Elimination as a public health problem	Yes	Yes	RDT needed for post-kala azar dermal leishmaniasis, leishmania skin test for mapping transmission
		RDT needed for East Africa		

Abbreviations: FTS, Filariasis test strip; IDA, ivermectin, diethylcarbamazine and albendazole; MDA, mass drug administration; M&E, monitoring and evaluation; POC, point of care; RDT, rapid diagnostic test; TAS, transmission assessment surveys.

## New scientific opportunities

Although the diagnostic needs for NTD programmes are extensive, there is reason to be optimistic about the prospects of development of new tools to address programme requirements. The past 20 y have seen an explosion in the development of new molecular biology methods, including rapid and low-cost genome sequencing and new approaches to analyse proteomes and secretomes. This technology has been applied to several NTDs, leading to identification of potential new biomarkers and, in some cases, tests that are already being evaluated in the laboratory and in the field. For example, several approaches are being employed to identify targets for onchocerciasis serological assays to support programmatic decision-making.^8–11^ These efforts should be expanded to include other NTDs. In particular, promising new epitope screening technologies may help to accelerate the identification of novel targets and permutations of targets for serological assays.^12^

In principle, the development of nucleic acid tests for diagnosis should be ‘low hanging fruits’; however, the lack of laboratory capacity to run molecular tests and the lack of standardisation of test formats have prevented test deployment on the scale needed by most programmes. These challenges must be addressed by development of either laboratory capacity, potentially through leveraging laboratory capacity for other diseases such as HIV and TB, or development of tests that do not require laboratory infrastructure for their performance. Test standardisation is necessary to ensure that treatment and programme decisions are based on consistent criteria.

Independent of the test format, once candidate assays are identified, more vigorous efforts to coordinate assay validation processes are needed to improve the rate at which new tests can be introduced into programmes. Certified reference materials will also be needed to evaluate test performance at individual laboratories once a new test has been rolled out. The COVID-19 pandemic has illustrated the relative speed at which new diagnostic tools can be developed when resources are abundant and collaborations are intensified. In the absence of new resources, the NTD community will have to create incentives for test developers by defining clear processes for regulatory approval and adoption of tests by programmes. In addition, advance purchase commitments and novel financing strategies should be considered to reduce the market risk for test manufacturers. As a more positive outcome of the pandemic, testing capacity at country level is expanding dramatically. This will create new opportunities to introduce molecular testing in settings where this capacity has been limited.

## Moving the agenda forward

Reaching the end game presents new challenges for NTD programmes as infection prevalence declines and incident cases become increasingly rare. Surveillance is a requirement to support claims that a disease has been eliminated or eradicated, but disease-specific surveillance strategies become harder to justify when resources are limited and active interventions have stopped. Multi-disease surveillance makes sense, in principle, but has been challenging to achieve in practice because of differing disease geographies, target populations, test formats and the traditionally vertical nature of some programmes. Although integrated serosurveillance is finding increased use as a research tool, the assay platforms typically require laboratory infrastructure and are not well standardised among laboratories.^13–15^ Innovative technologies that will support low-cost and flexible multi-disease surveillance are needed. Where programme decisions are made at the population level, surveys and survey design become an integral part of diagnosis; therefore, it is critical to capitalise on the use of new geostatistical tools that can lead to more efficient survey designs.^16^

As noted above, an essential role for the DTAG is to help provide clarity, both to test developers and donors on the critical needs of NTD programmes. Disease-specific subgroups have been created to develop detailed descriptions of the programmatic use case to provide useful context and guidance for scientists and product developers working to support the 2021–2030 NTD roadmap, with a particular emphasis on the generation of detailed TPPs to guide test development. Once drafted, TPPs will be posted on the WHO's Global Observatory on Health Research and Development for a 28-d period of public consultation prior to finalisation.

Cross-cutting groups are under development to address a range of issues common across NTDs, including:

- Surveillance platforms.
- Clinical diagnosis, imaging and microscopy.
- Manufacturing and regulatory pathways.
- Resource mobilisation.

The surveillance subgroup will focus on survey design, assay technology and tool adaptation for the surveillance setting. The clinical diagnosis, imaging and microscopy group will address topics such as improved training methodologies and materials, the criteria for evaluating diagnostic image analysis and machine learning as well as access issues limiting the availability of image libraries. The manufacturing and regulatory pathways will be charged with developing innovative strategies for overcoming barriers to test availability due to the absence of a large global market (i.e. extended development timelines and high unit costs). This subgroup will serve as the crucial link between the disease-specific subgroups and product developers, both those new and familiar to the NTD field, to ensure the translation of TPPs into real diagnostic tools capable of serving the needs of the programmes. The group will also work with the WHO to understand and fulfil regulatory requirements as new tools progress through the development pipeline. The resource mobilisation subgroup will work to improve coordination among existing donors, engage new donors and strengthen links between the WHO and the NTD diagnostics community. New cross-cutting subgroups may be established as the needs of the DTAG evolve.

As the work of the initial subgroups has progressed, additional topics have emerged with implications for multiple diseases. The operational challenge of test validation when trying to achieve very high sensitivity and specificity requirements has been raised as a subject for future strategising by test developers. For example, to demonstrate a 99.7% diagnostic specificity (discussed in the context of a hypothetical immunoassay), thousands of serum samples of sufficient volume would be needed for validation from across representative geographies, with and without various important coinfections, especially when confronted with the realities of laboratory validation such as variances between test lots. Similarly, several subgroups have raised the challenge of estimating sensitivity and specificity in the absence of a gold standard. Without a gold standard, true disease prevalence is unknown, and therefore sensitivity and specificity cannot be calculated directly. The use of statistical methods, such as latent class analysis, to overcome the absence of a gold standard, has been raised as a topic for cross-disease consideration.^17–19^

Throughout the development from TPPs to new product development, the DTAG and its subgroups will rely on well-accepted field principles to guide activities. The TPP template(s) populated or reviewed by subgroups will be based on the REASSURED criteria for diagnostic use in resource-limited settings: Real-time connectivity, Ease of specimen collection, Affordable, Sensitive, Specific, User-friendly, Rapid and robust, Equipment-free and environmentally friendly and Delivered to those in need.^20^ Such principles and tools will help to ensure that future tools are developed based on the needs and realities of NTD programmes.

### Conclusion

A robust DTAG will create greater awareness of the needs of NTD programmes and align investments, both financial and scientific, with those needs. Over the long term, the DTAG will play a more significant role in fostering innovation in the development of novel multiplex testing platforms and integrated surveys/screening.

## Data Availability

None.
